# Comparative genomics reveals putative copper tolerance genes in a *Fusarium oxysporum* strain

**DOI:** 10.1093/g3journal/jkae272

**Published:** 2024-11-19

**Authors:** Lorenz Rhuel P Ragasa, Christina A Cuomo, Ricardo C H del Rosario, Michael C Velarde

**Affiliations:** Institute of Biology, College of Science, University of the Philippines Diliman, Quezon City 1101, Philippines; Infectious Disease and Microbiome Program, Broad Institute of MIT and Harvard, Cambridge, MA 02142, USA; Stanley Center for Psychiatric Research, Broad Institute of MIT and Harvard, Cambridge, MA 02142, USA; Institute of Biology, College of Science, University of the Philippines Diliman, Quezon City 1101, Philippines; Natural Sciences Research Institute, College of Science, University of the Philippines Diliman, Quezon City 1101, Philippines

**Keywords:** copper-tolerant fungi, accessory chromosome, genome assembly

## Abstract

Copper has been widely used as a main component in fungicides due to its versatility and effectivity. However, copper contamination from the environment creates selective pressure for the emergence of copper-tolerant pathogenic fungal strains that may proliferate and further cause damage to important agricultural crops. Although some studies focused on specific cellular mechanisms of copper tolerance, comprehensive genomic data are lacking. Here, we examined the genes potentially involved in copper tolerance by conducting a comparative analysis of newly sequenced genomes of 2 *Fusarium oxysporum* strains, IB-SN1W (copper-tolerant) and Foc-3429 (copper-sensitive), with other *Fusarium* species. Whole-genome assembly and annotation identified 10 core chromosomes shared between the 2 strains. Protein prediction revealed 16,894 and 15,420 protein-coding genes for IB-SN1W and Foc-3429, respectively. There are 388 unique genes in IB-SN1W not found in Foc-3429, potentially contributing to copper tolerance. Furthermore, the identification of synteny between the 2 strains, including the analysis of orthologous genes within the *Fusarium* genus, confirmed the presence of accessory chromosomes that are specific to IB-SN1W, accounting for 13% of the genome. These accessory chromosomes consist of genes associated with cation transporter activity, vacuole, copper oxidases, and copper transporters which shed light on the potential mechanism of copper tolerance in this strain. Additionally, a region within an accessory chromosome contains a high density of copper-related genes, raising the possibility that horizontal transfer of these chromosomes may contribute to copper tolerance.

## Introduction

Organisms have evolved mechanisms to control copper levels for proper cellular function, but an excessively high amount of copper is detrimental to most organisms (Cervantes and Gutierrez-Corona 1994; Gaetke and Chow 2003; Borchard *et al*. 2018). Indeed, copper has been widely used as a fungicide due to its wide availability, low cost, and effectivity (Lamichhane *et al*. 2015). However, there are already numerous reports about the development of copper resistance in plant pathogenic fungi (Al-Daoude *et al*. 2009; Behlau *et al*. 2011; Tu *et al*. 2018; Raffa *et al*. 2019). This makes copper-based fungicides ineffective, allowing copper-resistant fungal pathogens to cause damage to crops.

Cellular copper homeostasis is primarily regulated by transcription factors that modulate the expression of a variety of genes when exposed to high or low amounts of copper (Song *et al*. 2019). These homeostasis mechanisms include cellular trafficking, storage, and detoxification (Antsotegi-Uskola *et al*. 2020). In addition to these homeostasis mechanisms, other survival strategies may also evolve within a cell to be able to tolerate high copper and minimize cell damage during high levels of copper. Copper tolerance mechanisms include metal solubilization, cell wall binding, enhanced efflux, intracellular chelation, subcellular compartmentalization, and antioxidant response (Cervantes and Gutierrez-Corona 1994; Cavalcanti Luna *et al*. 2015).

Environmental conditions influence the composition of microbial communities within host plants (Glynou *et al*. 2016). It can be expected that a high copper environment would favor the proliferation of copper-tolerant strains. Indeed, certain strains of many fungal species have already been identified for their ability to withstand high copper concentrations (Iskandar *et al*. 2011; Feng *et al*. 2017; Tu *et al*. 2018; Dias *et al*. 2019). In our previous work, a mixed population of *Fusarium* sp. from a *Solanum nigrum* L. plant, growing at a mine tailing of a gold extraction facility, led to the selection of a highly copper-tolerant *Fusarium oxysporum* strain after exposure to 250 ppm copper (Ragasa *et al*. 2021). Since there is a need for a comprehensive investigation of genes involved in copper tolerance, we assembled and examined the draft genome of the copper-tolerant *F. oxysporum* strain in the current study and compared it to the genome of a copper-sensitive strain. Given that *Fusarium* genomes contain accessory chromosomes (lineage-specific chromosomes) which may contain important genetic information involved in the pathogenicity and stress tolerance within a specific host (Ma *et al*. 2010), we also conducted comparative genome analysis of these 2 strains relative to a reference strain to determine core and accessory chromosomes, while comparison with different *Fusarium* species determined genes that are unique or shared across other *Fusarium* species.

## Materials and methods

### Whole-genome extraction, quality control, and sequencing

*F. oxysporum* IB-SN1W strain came from our previous study (Ragasa *et al*. 2021) while Foc-3429 strain was obtained from the Philippine National Collection of Microorganisms (PNCM), BIOTECH, University of the Philippines Los Baños. Fungal cultures were grown on potato dextrose agar (PDA) with either no copper or 300 ppm copper. Sterilized PVDF membrane was briefly soaked in potato dextrose broth with corresponding treatment, then placed on top of a semisolid PDA plate. Fungal spores were spread directly on top of the equilibrized PVDF membrane in agar. After 5 days, hyphae were carefully scraped off the membrane for DNA extraction using Zymo fungal miniprep according to the manufacturer's protocol. Extracted DNA was assessed using agarose gel electrophoresis, Nanodrop spectrophotometry, and Qubit fluorometry. High-quality samples (OD260/280∼1.91-1.92, >18 µg, >100 ng/µL) were sent to 1st BASE (Singapore) for sequencing using PacBio Sequel II with CLR mode.

### Assembly and annotation

Reads were assembled using Canu 2.2 (Koren *et al*. 2017) with default parameters using CLR mode with correction, trimming, and assembly. Genome assessment was performed using Benchmarking Universal Single-Copy Orthologs (BUSCO, v5.3.2) (Manni *et al*. 2021) in genome mode using eukaryota, fungi, and hypocreales gene sets. Gene annotation was performed using protein homology to improve gene calling and structure predictions. Briefly, a database of available proteins was created from high-quality *Fusarium* genomes available in NCBI [*Fusarium solani-FSSC-5* (GCA_020744495.1), *F. solani-melongenae* (GCA_023101225.1), *Fusarium ambrosium* (GCA_003947045.1), *Fusarium euwallaceae* (GCA_003957675.1), *Fusarium duplospermum* (GCA_003946985.1), *Fusarium pseudograminearum* (GCA_000303195.2), *Fusarium graminearum* (GCA_000240135.3), *Fusarium oxysporum lycopersici 4287* (GCA_000149955.2), *F. oxysporum Fo47* (GCA_013085055.1), and *Fusarium fujikuroi* (GCA_900079805.1)].This database was used to predict genes using BRAKER2 v2.1.6 (Brůna *et al*. 2021) using the –fungus option. tRNAs were predicted using tRNAscan-SE (Chan and Lowe 2019) and rRNAs predicted using RNAmmer 1.2 (Lagesen *et al*. 2007). Genes containing PFAM domains found in repetitive elements or overlapping tRNA/rRNA features were removed. Genes were named and numbered sequentially. Repeat elements were determined using RepeatModeler 2.0 (Flynn *et al*. 2020). For enrichment analysis, genes were functionally annotated by assigning PFAM domains, Gene Ontology (GO) terms, and KEGG classification using eggNOG-mapper 2.1.9 (Cantalapiedra *et al*. 2021). For subsequent analyses, copper-related genes were defined as genes associated with pfam and GO terms that contains the term “copper” in the name.

### Comparative genome analysis

Comparative genome analysis to determine the differences and similarities between the genomes, the assemblies were compared to other closely related *Fusarium* species. The assembly was first aligned to a reference assembly of *F. oxysporum* f. sp. *lycopersici* 4287 (GCA_000149955.2; ASM14995v2) using nucmer and visualized using mummerplot (mummer4) (Marçais *et al*. 2018). The assemblies were also aligned to each other. Synteny plot was created using RIdeogram (Hao *et al.* 2020) showing only those alignments with a total of at least 200 kb alignment between 2 contigs. To determine orthologs, OrthoFinder v2.5.4 (Emms and Kelly 2019) was used to compare the genomes with other *Fusarium* species (same accessions as the ones used for BRAKER). The number of shared orthologs were then determined and visualized using an upset plot (Conway *et al.* 2017) in RStudio. To determine the presence of selected copper-related genes, top blast hit species of copper-related genes in IB-SN1W with whole-genome assemblies were determined, and annotated peptide sequences were obtained if available (Supplementary Table 1). OrthoFinder was used to compare these genomes and generate gene trees and a species tree.

### Gene enrichment

Gene enrichment was performed on genes unique in IB-SN1W, or genes in accessory chromosomes, with all genes in all chromosomes as background. Fisher's exact test was used with Bonferroni correction.

## Results

### Genome assembly and annotation

Genome sequencing using PacBio Sequel II CLR library generated a total of 23.88 Gb and 21.83 Gb for the copper-tolerant IB-SN1W and copper-sensitive Foc-3429, respectively. A total of 2.5 and 3.1 M unfiltered subreads were generated with an N50 of 9,935 and 8,814 bases, respectively. Although this is less than the typical N50 of subreads (Warmington *et al*. 2019), this represents about 420× genome coverage. Genome assembly produced a total genome size of 49.05 Mb and 45.54 Mb representing 60 and 149 contigs with an average length of 817 and 305 kb, respectively. Genome assembly assessment by BUSCO (v5.3.2) revealed high number of conserved genes (>99%) for all BUSCO data sets used, supporting the completeness of the assemblies (Supplementary Fig. 1).

**Fig. 1.**
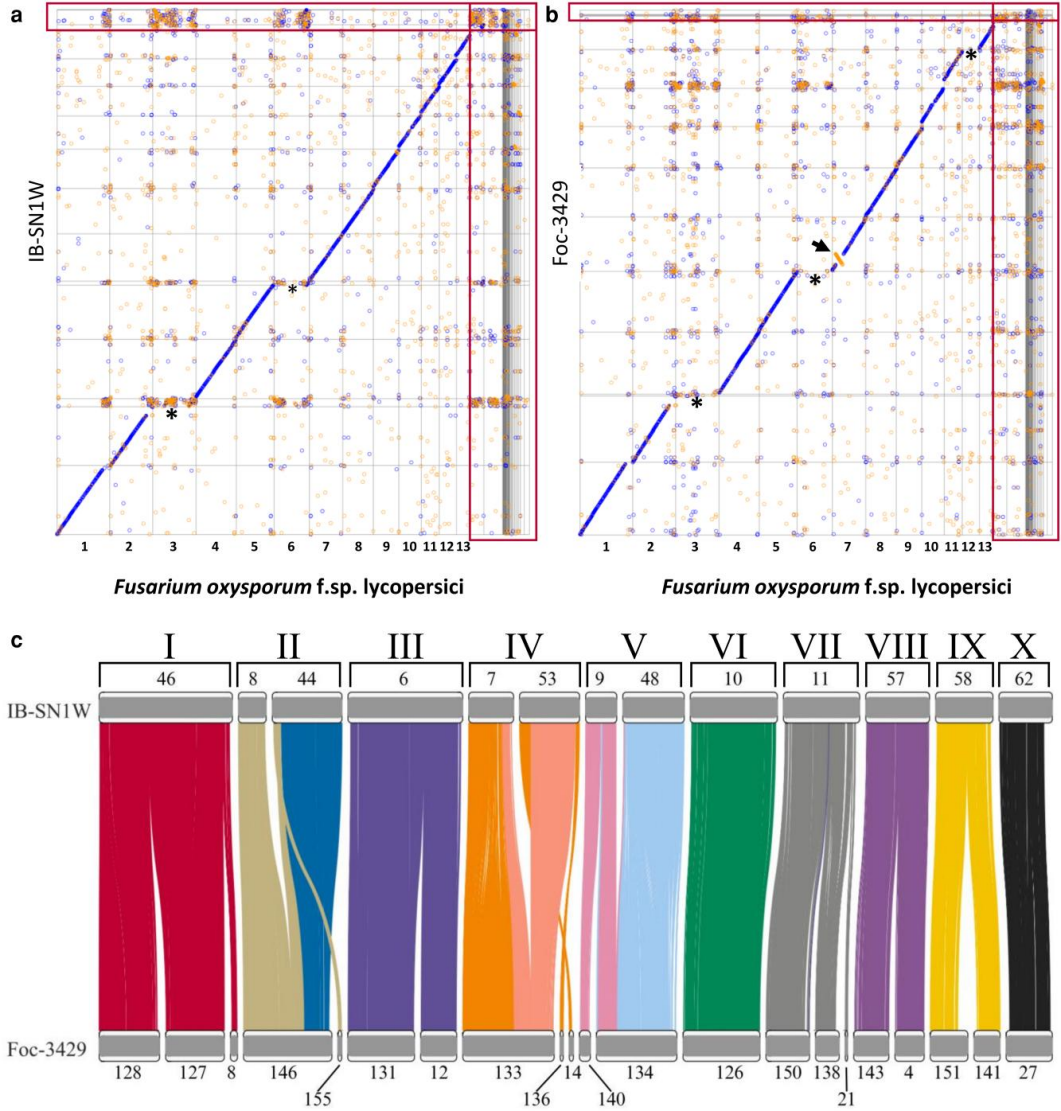
Whole-genome assembly reveals 10 core chromosomes in IB-SN1W and Foc-3429. Nucmer alignment of IB-SN1W a) and Foc-3429 b) with *F. oxysporum* f. sp. *lycopersici* 4287 as reference. Asterisks denote the missing chromosomes compared to the reference, while the arrow shows the inversion at chromosome 7 in Foc-3429. Accessory chromosomes are bound within the boxes. c) Synteny of chromosomes between IB-SN1W and Foc-3429 showing alignment between contigs with a total of at least 200 kb alignment. Numbers indicate the assembly contig number, while the roman numerals represent the putative chromosome number.

Whole genomes of the 2 strains were further aligned with the reference genome *F. oxysporum* f. sp. *lycopersici* 4287 (Fol 4287), which is the most annotated genome phylogenetically closest to the 2 strains, to help identify notable genetic variants between these strains. Whole-genome alignment of the assembled contigs using Nucmer showed a notable inversion within chromosome 7 in Foc-3429 but not in IB-SN1W (Fig. 1, a and b). Additionally, chromosomes 3 and 6 are not present in both strains, while chromosome 12 is present in IB-SN1W but not in Foc-3429. Hence, core chromosomes were then identified as contigs that are present in both strains. Contigs not found in these core chromosomes were assigned as accessory contigs which form parts of the accessory chromosomes. These consisted of 13 and 5% of the genome for IB-SN1W and Foc-3429, respectively. There were at least 10 core chromosomes in IB-SN1W and Foc-3429 which were named sequentially based on their alignment with the reference assembly. This allowed us to determine synteny of the 2 assembled genomes (Fig. 1c).

The IB-SN1W genome contained a relatively small percentage of repetitive DNA sequence at 1.01%. Previous reports showed that *Fusarium* species have more than 2% repeats (Hao *et al*. 2021; Kuang *et al*. 2022), while other species such as *F. oxysporum* f. sp. *Lini* (Krasnov *et al*. 2020) and *F. fujikuroi* F250 (Bashyal *et al*. 2017) have less than 0.7% repeats. Core chromosomes of IB-SN1W contained 0.7% repeats while accessory chromosomes were consisted of 2.8% repeats. Some regions were also enriched with repeats, which may contribute to further genetic variation by chromosomal rearrangements. Protein prediction resulted in 16,894 and 15,420 protein-coding genes for IB-SN1W and Foc-3429, respectively. Of these, 15,409 (91.2%) and 14,277 (92.58%) were annotated with GO terms, KEGG pathways, and PFAM domains using eggnog. IB-SN1W has 2,150 (12.7%) genes in accessory chromosomes, while Foc-3429 has 528 (3.42%).

### Comparative genome analysis

**Fig. 2.**
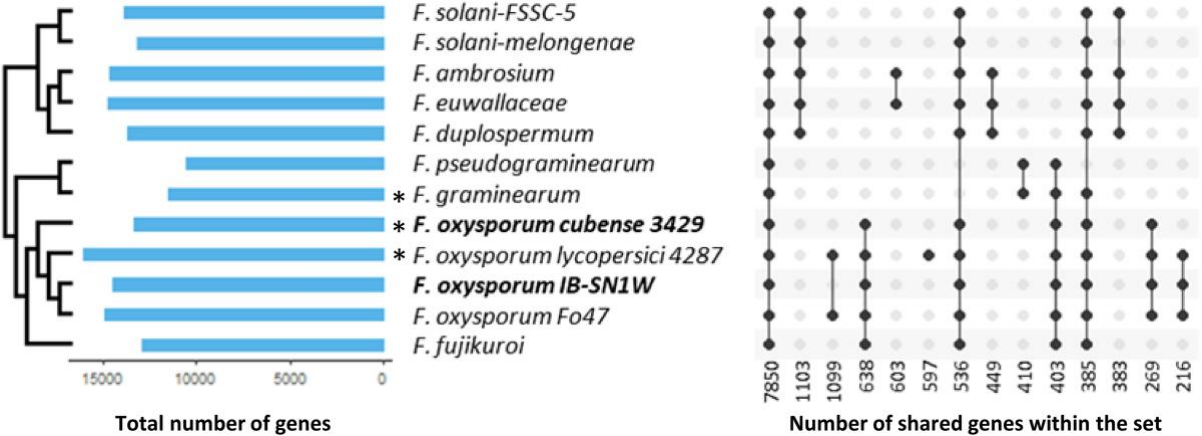
Orthologous gene families are shared between *Fusarium* species. The upset plot shows intersection of gene families that are shared across different *Fusarium* species. Orthologs were determined using OrthoFinder. Phylogeny is based on species tree of orthologous genes. Asterisks denote the strains known to be copper sensitive.

To determine unique genes which may confer copper tolerance and genes shared across species, we compared the genome assemblies of IB-SN1W and Foc-3429 with high-quality assemblies from different animal and plant pathogenic *Fusarium* species belonging to *F. solani* and *F. oxysporum* species complexes (Fig. 2). Among these, *F. oxysporum* f. sp. *lycopersici* and *F. graminearum* are confirmed to have low copper tolerance (Liu *et al*. 2020; Lorenzo-Gutiérrez *et al*. 2020). A total of 21,768 gene families were identified across all *Fusarium* species using OrthoFinder, with 7,850 (36%) gene families shared in all species. *F. oxysporum* strains shared 269 species-specific gene families, while the copper-tolerant IB-SN1W strain contained 388 genes without shared families. Based on the species tree inference of OrthoFinder from orthologous genes, IB-SN1W was more closely related to Fo47 than any other *F. oxysporum* strains analyzed, with Foc-3429 being the most distant strain.

**Fig. 3.**
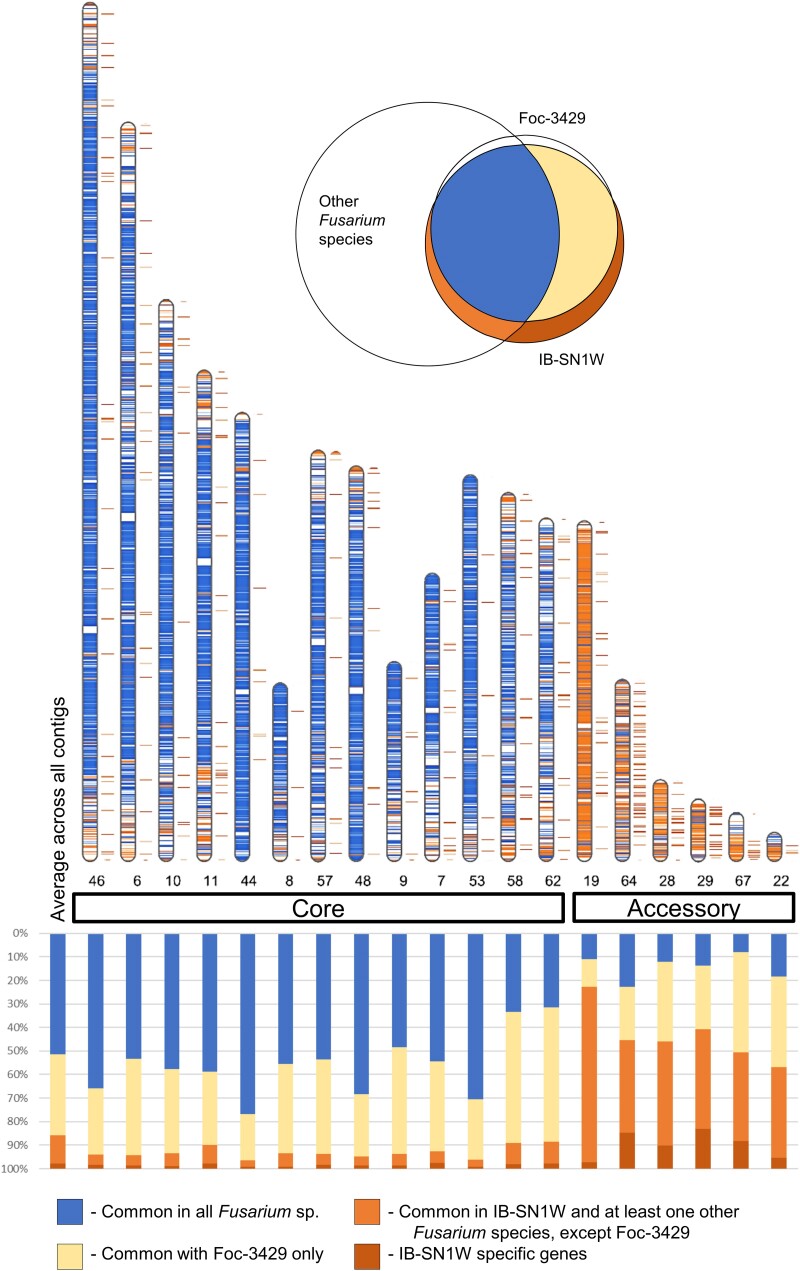
Accessory chromosomes in IB-SN1W consist mostly of unique genes compared to other *Fusarium* species. Shown in the karyogram are all the assembled contigs which represent core chromosomes. Only the top 6 longest contigs assigned as accessory chromosomes are shown. The graph below shows proportion of genes common in all *Fusarium* included, common with Foc-3429 only, present in IB-SN1W and all *Fusarium* included but not in Foc-3429, and genes found only in IB-SN1W. The Venn diagram represents gene assignments based on intersections between species.

To aid in delineating core and accessory chromosomes by synteny, we mapped genes that were shared among all *Fusarium* species alongside strain-specific genes within the contigs of IB-SN1W. Strain-specific genes were defined as genes found exclusively in IB-SN1W, but not in Foc-3429, or any other *Fusarium* genome included in the study. In this analysis, contigs in the core chromosomes of IB-SN1W had more *Fusarium*-common genes and less strain-dependent genes than contigs in its accessory chromosomes (Fig. 3). Core contigs range from 1.3 to 6.4 Mb in length while the 6 largest accessory contigs range from 0.2 to 2.5 Mb in length (Supplementary Data 1). Core contigs contain only 2.6 to 8.3 strain-specific genes per kilobase, while the largest accessory contigs contain a higher density of strain-specific genes at 10.6 to 53.6 genes per kilobase. Most notably, accessory contig 19 had the highest number of genes unique to the copper-tolerant IB-SN1W and at least 1 other *Fusarium* species but absent in the copper-sensitive Foc-3429. Indeed, accessory chromosomes of IB-SN1W consist mostly of strain-specific genes and are smaller in size, which is consistent with properties of accessory chromosomes in other fungi (Galazka and Freitag 2014).

### Identification of copper tolerance-related genes

**Fig. 4.**
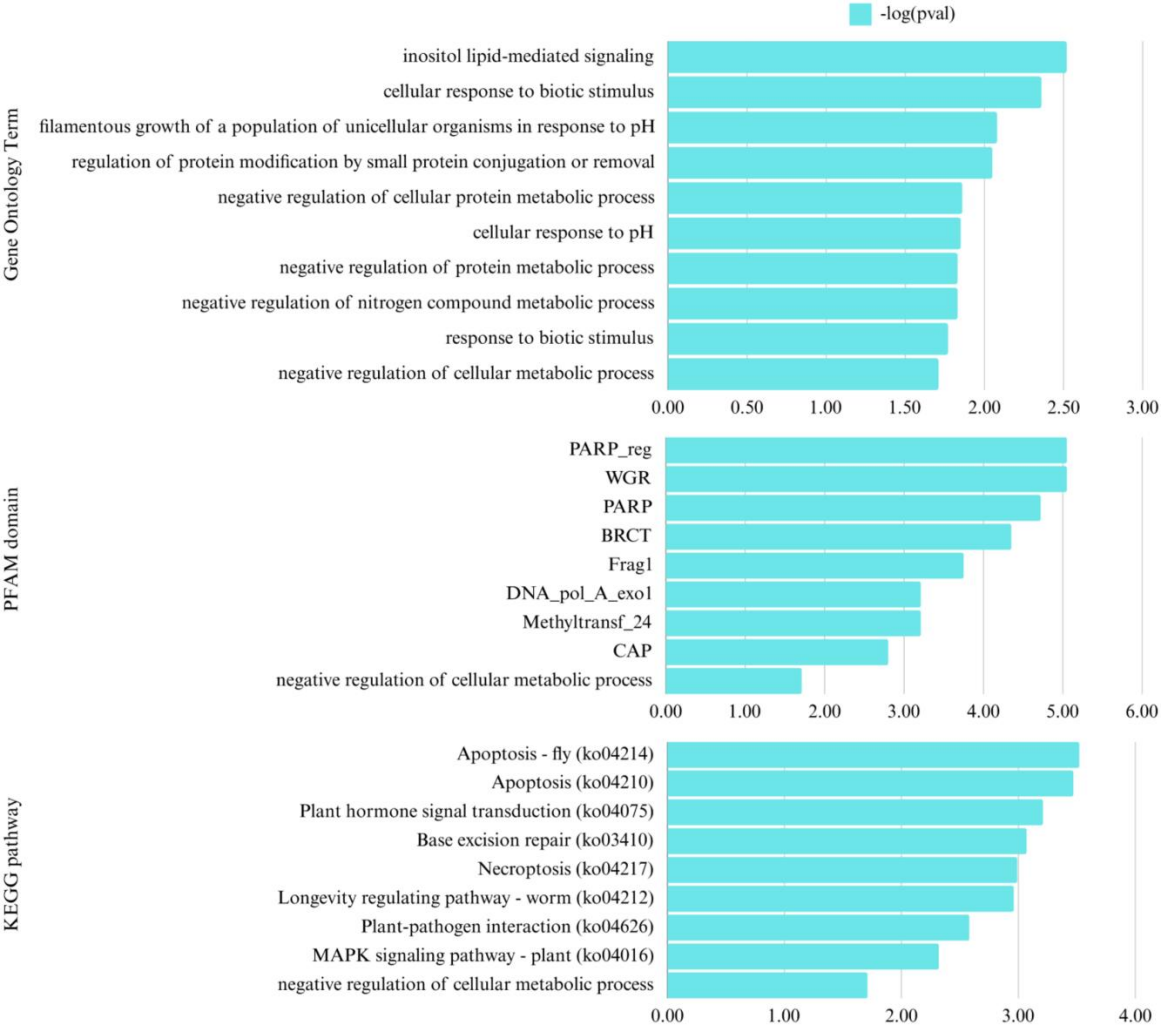
Unique genes in IB-SN1W are enriched genes involved in cellular repair. GO, PFAM, and KEGG terms are shown with their corresponding *P*-value and log2 fold change in the number of genes relative to the whole genome.

*F. oxysporum* f. sp. *Lycopersici* 4287 has been reported to tolerate less than 250 ppm copper (Lorenzo-Gutiérrez *et al*. 2020), indicating the absence of genes involved in copper tolerance. Similarly, the copper-sensitive Foc-3429 strain would also unlikely contain such genes. Hence, some of the unique genes present in IB-SN1W may be associated with its copper tolerance and may include putative copper tolerance genes. To identify potential genes that may be involved in copper tolerance, we identified and analyzed IB-SN1W-specific genes which were not found in Foc-3429 genome (Fig. 4; Supplementary Data 2 and 3). Using all IB-SN1W genes as background, an overrepresentation test of IB-SN1W-specific genes revealed that these were most significantly enriched for several genes involved in inositol lipid-mediated signaling, cellular response to biotic stimulus, and negative regulation of metabolic processes. IB-SN1W-specific genes also included DNA damage repair genes, such as poly (ADP-ribose) polymerase (PARP) and BRCT (BRCA1 C-Terminus) domains (Fig. 4; Supplementary Data 4).

**Fig. 5.**
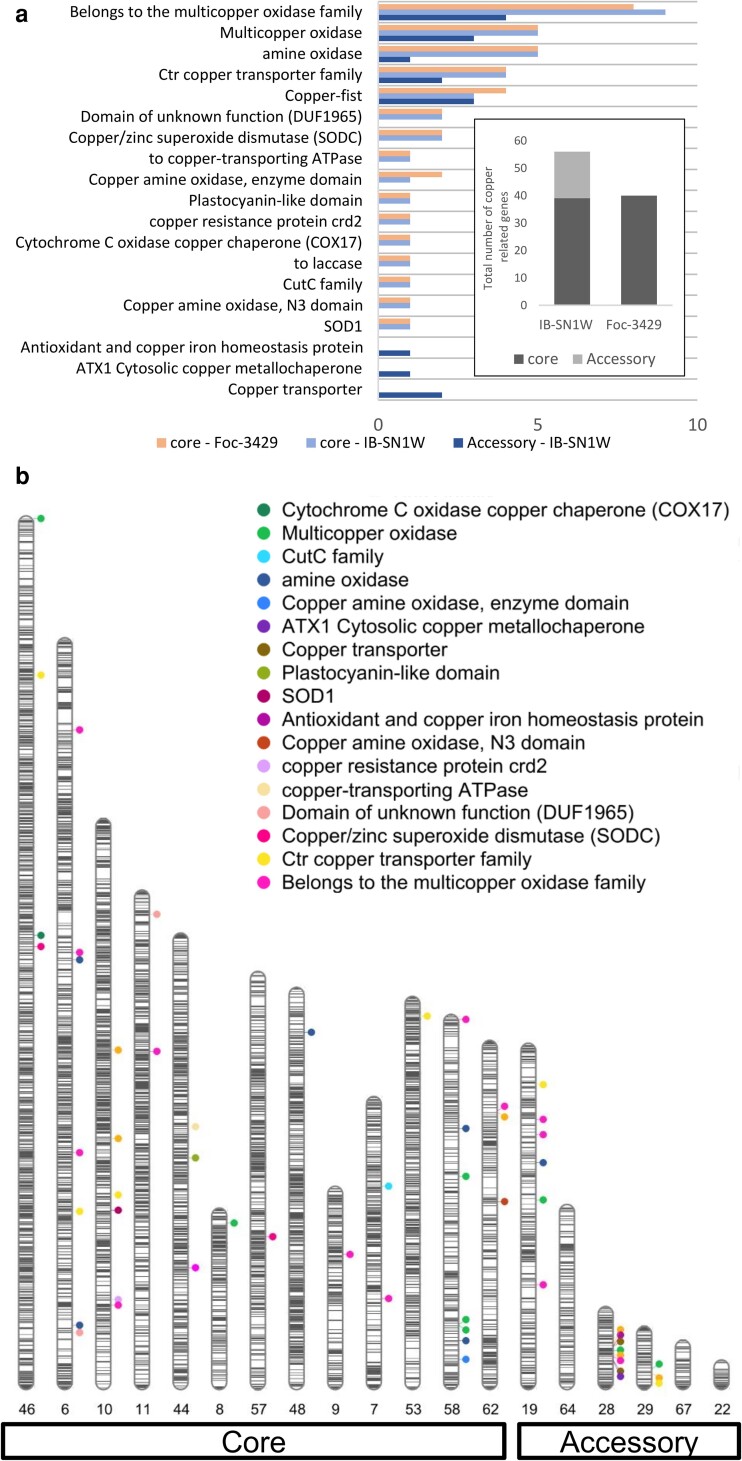
Several copper-related genes are found in the accessory chromosome of IB-SN1W but not in Foc-3429. a) Copper-related genes are selected from the annotated genes, and their chromosomal locations are shown. Inset shows that both IB-SN1W and Foc-3429 have around 40 copper-related genes in core chromosomes, while IB-SN1W has an additional 17 genes in the accessory chromosome. Among these are copper oxidases and copper transporters. b) Repeat elements and copper-related genes. Bands show repeat elements, which consist of 1.03% of the genome. Circles indicate the location of various copper-related genes. Most notably, contig 28 has the highest number of copper-related genes.

Overrepresentation test on genes of the accessory chromosomes of IB-SN1W showed that these were enriched in genes involved in cation transporter activities, most notably transmembrane transport, and antiporter activity (Table 1). Genes involved in lytic and storage vacuoles were also enriched. While the core chromosomes of IB-SN1W and Foc-3429 shared several similar copper-related genes, the accessory chromosomes of Foc-3429 did not contain any copper-related genes (Fig. 5a). IB-SN1W contained an extra 17 copper-related genes in its accessory chromosomes, with contig 28 having the greatest number at 8 genes (Fig. 5b).

**Table 1.** Top enriched GO terms in accessory chromosomes of IB-SN1W.

GO term	*P*-value^a^	*P*adj^a^	# of genes
Di-, trivalent inorganic cation transmembrane transporter activity	1.99E-07	1.19E-04	13
Hydrolase activity, hydrolyzing O-glycosyl compounds	2.19E-07	1.19E-04	12
Secondary active transmembrane transporter activity	4.58E-07	1.23E-04	13
Antiporter activity	4.66E-07	1.23E-04	9
Metal ion transmembrane transporter activity	8.49E-07	1.80E-04	13
Hydrolase activity, acting on glycosyl bonds	1.74E-06	3.28E-04	12
Lytic vacuole	5.23E-06	8.28E-04	26
Calcium:hydrogen antiporter activity	6.33E-06	8.28E-04	5
Potassium:hydrogen antiporter activity	1.13E-05	1.13E-03	5
Mitochondria-nucleus signaling pathway	1.13E-05	1.13E-03	5
Anion transmembrane transporter activity	1.67E-05	1.57E-03	20
Ion transmembrane transporter activity	1.76E-05	1.58E-03	21
Polysaccharide catabolic process	2.27E-05	1.78E-03	9
Phosphate transmembrane transporter activity	2.63E-05	1.78E-03	19
Inorganic solute uptake transmembrane transporter activity	2.63E-05	1.78E-03	19
Transition metal ion transport	3.14E-05	1.78E-03	9
Cobalt ion transport	3.04E-05	1.78E-03	4
Storage vacuole	4.01E-05	2.13E-03	24
Regulation of flocculation	5.93E-05	2.75E-03	4
Glycerol-1-phosphatase activity	5.93E-05	2.75E-03	4

^a^Fisher’s exact test with Bonferroni correction.

**Fig. 6.**
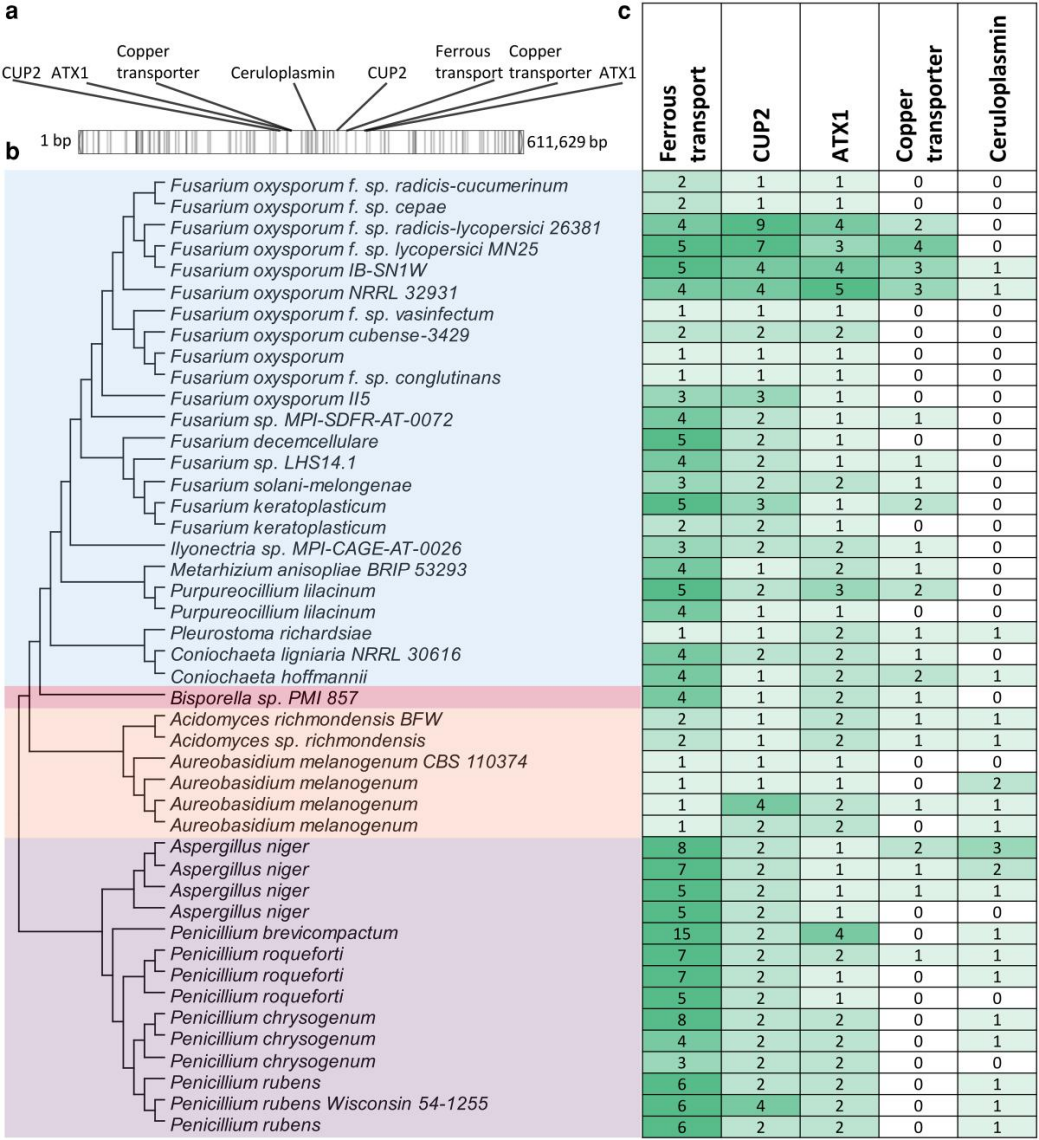
Distribution of orthologs of copper-related genes in IB-SN1W contig 28 across ascomycetes. a) Zoomed in view of contig 28 showing the location of copper-related genes and repeats (bands). b) Species tree of selected ascomycetes containing copper-related gene orthologs from orthofinder, grouped according to taxonomic classes (top to bottom: Sordariomycetes, Leotiomycetes, Dothideomycetes, Eurotiomycetes). c) Number of orthologs of copper-related genes per species.

Further investigation of genes in contig 28 of IB-SN1W revealed enrichment of genes involved in metal ion transport and homeostasis (Table 2) with 8 genes that belong to the copper oxidase family and copper transporters located within 115 kb in the middle of the contig. Among these are duplicated copies of genes coding for copper metallochaperone Atx1 (FOIBW_007834, FOIBW_007870), copper binding transcription factor Cup2 (FOIBW_007828, FOIBW_007857), and an uncharacterized copper transporter (FOIBW_007835, FOIBW_007869). Atx1 transports Cu(I) to the endoplasmic reticulum, where it is prepared for export via the copper transporter ATPase Ccc2p (Lin *et al*. 1997). Cup2 is a transcription factor that activates metallothionein in response to high copper (Welch *et al*. 1989). Both of which attempt to detoxify copper via chelation and eventual export outside of the cell (Moraes *et al*. 2023). There is also a single copy of an iron transporter multicopper oxidase (FOIBW_007859) in contig 28, with another homolog in the core chromosome. Another multicopper oxidase identified is a ceruloplasmin homolog (FOIBW_007850) which requires copper for its primary function in iron transport in mammals (Hellman and Gitlin 2002). This gene is not found in other *Fusarium* species but is present in human opportunistic pathogen *F. oxysporum* NRRL32931. When compared to other ascomycetes, ceruloplasmin homologs can also be found in some species, particularly in Eurotiomycetes which includes the genus *Penicillium* and *Aspergillus* (Fig. 6). Moreover, contig 28 and its copper-related genes have no homology with other closely related species such as *F. oxysporum* lycopersici 4287 and *F. oxysporum* Fo47. Overall, despite different lineages, copper-related genes in contig 28 of IB-SN1W is highly homologous with genes in *F. oxysporum* NRRL32931 and with other ascomycetes (Supplementary Figs. 2–6), suggesting the possibility of horizontal gene transfer.

**Table 2.** Top enriched GO terms in contig 28 of IB-SN1W.

GO term	*P*-value	*P*adj	# of genes
Transition metal ion transport	3.3758E-9	1.2211E-6	7
Copper ion transport	1.5437E-8	1.2211E-6	5
Cobalt ion transport	1.6152E-8	1.2211E-6	4
Cellular cobalt ion homeostasis	1.6152E-8	1.2211E-6	4
Cobalt ion homeostasis	1.6152E-8	1.2211E-6	4
Cellular copper ion homeostasis	4.6849E-8	2.5299E-6	5
Copper ion homeostasis	4.6849E-8	2.5299E-6	5
Metal ion transport	3.7523E-7	1.7730E-5	7
Cellular cadmium ion homeostasis	1.0821E-6	3.7184E-5	3
Cadmium ion homeostasis	1.0821E-6	3.7184E-5	3

## Discussion

To date, only a few genomes of plant fungi associated with copper tolerance have been analyzed (Tang *et al*. 2012, 2013; Karunasekera *et al*. 2019; Urquhart *et al*. 2022). This paper is the first to report a direct comparison of the genome of a copper-tolerant *Fusarium* strain and a copper-sensitive plant pathogenic strain which allowed the identification of genes potentially involved in copper tolerance. Here, we demonstrated the presence of accessory chromosomes between the 2 strains comprising 5 and 13% of the length of Foc-3429 and IB-SN1W genomes, respectively. Strain-specific genes were found mostly in accessory chromosomes but were also present in core chromosomes and were mostly composed of genes involved in the suppression of metabolic processes and DNA damage repair. As lowering metabolic processes limits the production of possible toxic secondary metabolites following copper metabolism (Scheckhuber *et al*. 2012), this downregulation of metabolic processes in IB-SN1W could alleviate further cellular damage due to copper. In addition, the presence of genes encoding for proteins with PARP and BRCT domains, known to be involved in DNA damage repair (Morales *et al*. 2014; Peña-Guerrero *et al*. 2023), also suggests an enhanced ability of the fungus to repair damaged DNA, presumably caused by copper-induced oxidative stress (Gaetke *et al*. 2014). Lastly, copper-related genes, such as copper oxidases and copper transporters, were found in accessory chromosomes of IB-SN1W but not in Foc-3429.

Increase copper export is associated with copper tolerance in fungi by the expression of ATPases such as CrpA (*Aspergillus fumigatus*), CaCRP1 (*Candida albicans*), and FgCrpA (*F. graminearum*) (Weissman *et al*. 2000; Wiemann *et al*. 2017; Liu *et al*. 2020). Copper compartmentalization through transport also prevents copper from causing cellular damage (Cornejo *et al*. 2013). The abundance of copper transporter genes in IB-SN1W suggests their possible involvement in copper tolerance by transporting excess copper outside the cell for removal or into vacuoles to rendering them nontoxic. Experimental validation of these genes in future studies will then be essential in supporting their involvement in copper tolerance.

Another strategy for copper tolerance is copper detoxification. In *Escherichia coli*, a multicopper oxidase is involved in the detoxification of copper by oxidizing Cu(I) into the less toxic Cu(II) (Grass and Rensing 2001; Cortes *et al*. 2015). Interestingly, a multicopper oxidase ceruloplasmin homolog is also located in accessory chromosomes of a human pathogenic *F. oxysporum*, likely acquired through horizontal gene transfer from other organisms living in extreme environments (Zhang *et al*. 2020). Moreover, ceruloplasmin homologs are present in ascomycetes such as *Penicillium* and *Aspergillus*, which thrive in extreme environments (Leitão 2009; Rose and Devi 2018). Hence, the presence of a ceruplasmin homolog in IB-SN1W suggests a potential role of the gene in copper tolerance, albeit further experiments are needed to support this hypothesis.

Horizontal transfer of accessory chromosomes may result in the acquisition of genes associated with host specificity and pathogenicity (Ma *et al*. 2010). Although most accessory chromosomes are usually associated with host specificity, tolerance to abiotic stressors can also be attributed to the presence of accessory chromosomes (Wei *et al*. 2024). This could result in a rapid evolution of strains that are highly tolerant to fungicides such as copper, thus increasing their pathogenicity to plants. Indeed, horizontal transfer of accessory chromosomes can cause nonpathogenic strains to become pathogenic due to the acquisition of virulence from the acquired chromosome (Zhang *et al*. 2020). In this study, the accessory contig 28 which contains a high density of copper-related genes may be a result of horizontal transfer because of its strain specificity. This can potentially confer copper tolerance to the recipient fungi.

Although transfer of whole chromosome was not explored, genes that are potentially obtained via horizontal gene transfer were identified. Indeed, the unequal distribution of genes in this chromosome and its homologs across ascomycete fungi suggest horizontal transfer of these genes. This is particularly detrimental to the host plant in cases where a fungal community contains a pathogenic strain which develop copper tolerance. Since new pathogenic lineages may arise even from genetically distant species (Gale *et al*. 2003), it is possible for highly pathogenic strains such as Foc-3429, a known plant pathogen that causes *Fusarium* wilt in banana (Warman and Aitken 2018), to acquire copper tolerance from IB-SN1W if the 2 fungi would grow close to each other. This then highlights the need for crop management strategies such as regulation of use of fungicides to prevent the spread of antifungal-tolerant pathogenic fungi.

The unique copper-related genes in the copper-tolerant IB-SN1W may also contribute to the potential pathogenicity of this strain in humans, in addition to its role in copper tolerance. During fungal infection in humans, copper levels may increase at the site of infection, which creates a toxic environment to eliminate the pathogen (Samanovic *et al*. 2012; Ding *et al*. 2013). However, copper-tolerant pathogens can evade this copper toxicity by employing various components of copper homeostasis such as copper transporters and copper chelators as seen in *Cryptococcus neoformans* (Ding *et al*. 2011). Indeed, the regulation of copper homeostasis is important in the survival of opportunistic fungi in high copper microenvironment within the host during infection. Interestingly, the high similarity of copper-related genes in the human pathogenic *F. oxysporum* NRRL32931 with those in *F. oxysporum* IB-SN1W relative to other nonpathogenic strains suggests the potential pathogenicity of IB-SN1W in humans. Together with the potential for horizontal gene transfer, these data suggest that copper tolerance in fungi may also be an indication of potential opportunistic pathogenicity in humans.

It is important to note that copper starvation is another strategy to combat microbial pathogens. In the human infection of *Candida albicans*, serum ceruloplasmin is used as a copper source by the microbial pathogen during copper starvation (Besold *et al*. 2021). Since a gene encoding for a ceruloplasmin homolog is found in both the NRRL32931 and IB-SNW1 strains but not in other *Fusarium* species, it is tempting to speculate that both NRRL32931 and IB-SNW1 will also tolerate copper starvation, by using its own ceruloplasmin as a copper reserve. However, further studies are needed to explore this hypothesis.

In summary, we successfully generated a high-quality genome assembly of the copper-tolerant *F. oxysporum* IB-SN1W and identified strain-specific genes that might be related to copper tolerance. Moreover, unique copper-related genes, including those related in copper transport, are abundant in IB-SN1W, especially in its accessory chromosomes. In conjunction with the possibility of horizontal transfer of accessory chromosomes and genes among closely related species, these results may suggest that copper tolerance can potentially be acquired by highly pathogenic fungi. These findings contribute to understanding fungal adaptation to high copper environments and may inform strategies for managing the proliferation of copper-tolerant pathogenic fungi.

## Supplementary Material

jkae272_Supplementary_Data

## Data Availability

The genome sequencing reads and assembled genome are deposited in the NCBI under BioProject PRJNA1100449 and BioSamples SAMN40969015 (IB-SN1W) and SAMN40969016 (Foc-3429). Raw reads are under accessions SRR28675651 and SRR28675650, while assembled genomes are under accessions JBCLMO000000000.1 and JBCLMP000000000.1 for IB-SN1W and Foc-3429, respectively. The assembled genome files are available at FigShare: https://doi.org/10.25387/g3.25893463. Custom scripts were not used in the assembly process, and all methods are described within the article. Supplemental material available at G3 online.
