# Supplementary material for: Comparative genomics reveals putative copper tolerance genes in a Fusarium oxysporum strain
Source: G3 (Bethesda). 2024 Nov 19;15(1):jkae272. doi: 10.1093/g3journal/jkae272 (PMC11708227; doi:10.1093/g3journal/jkae272)
Supplement: jkae272_Supplementary_Data [file jkae272_supplementary_data.zip › Supplementary_Figures_and_Tables_G3-2024-405225.pdf]

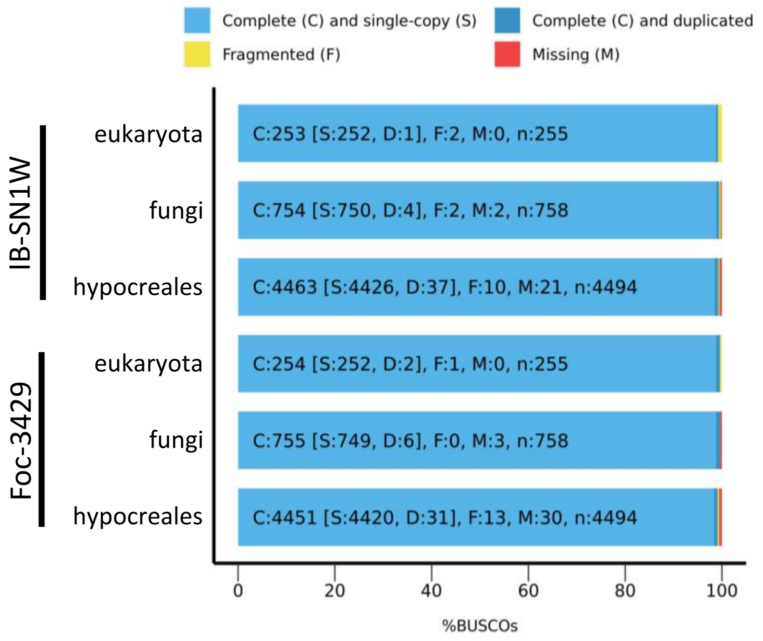

Supplementary Figure 1. Pacbio assembly and annotation indicate good genome completeness. BUSCO's were assessed using eukaryota, fungi, and hypocreales database. Over 99% of the complete and single-copy BUSCOs were found in the genome, while there are no missing BUSCOs from the eukaryota database

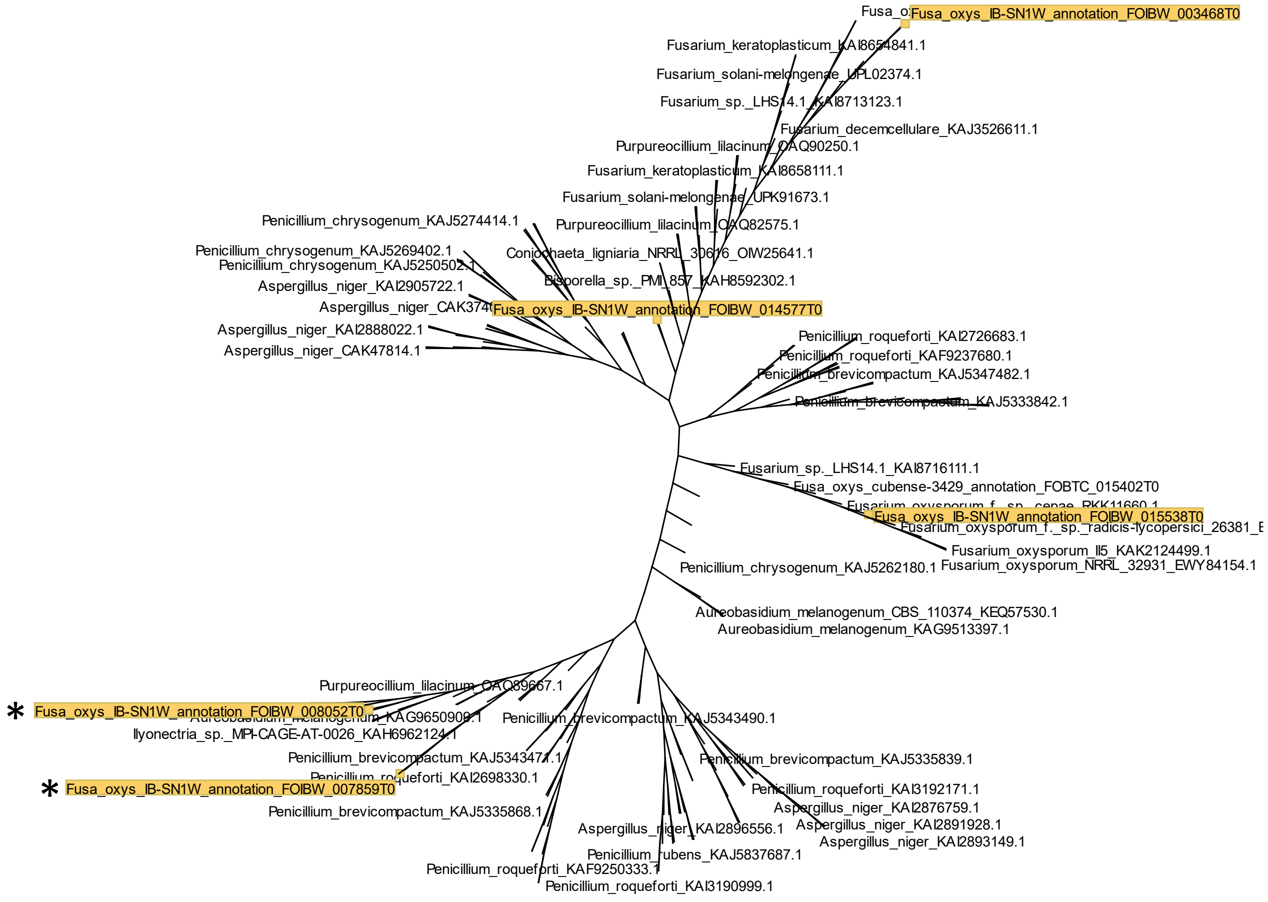

Supplementary Figure 2. Phylogenetic tree of ferrous transporter homologs. Highlighted in yellow are peptides from IB-SN1W. Asterisks denote its localization in the accessory chromosome

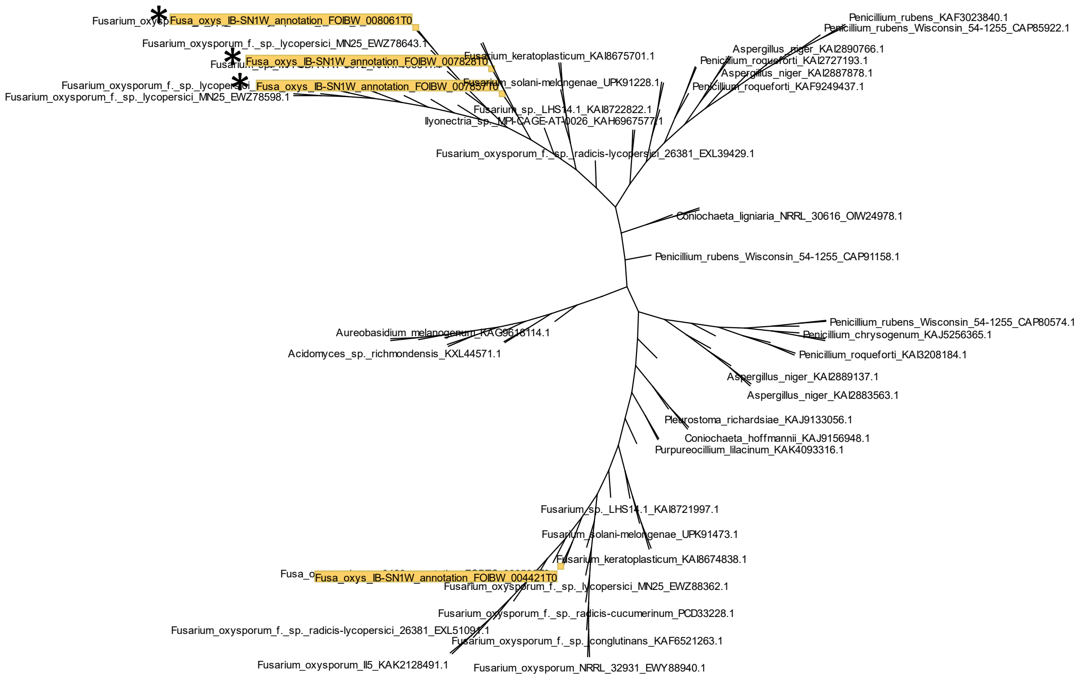

Supplementary Figure 3. Phylogenetic tree of CUP2 homologs. Highlighted in yellow are peptides from IB-SN1W. Asterisks denote its localization in the accessory chromosome

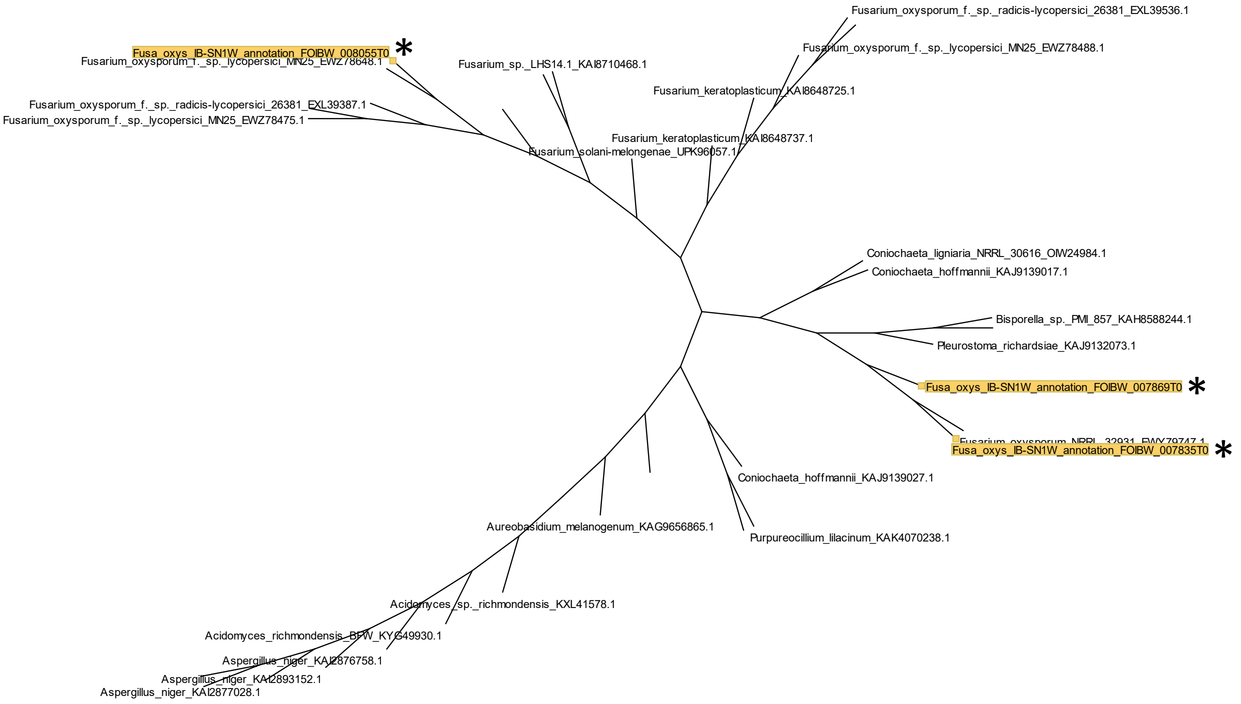

Supplementary Figure 4. Phylogenetic tree of uncharacterized copper transporter homologs. Highlighted in yellow are peptides from IB-SN1W. Asterisks denote its localization in the accessory chromosome

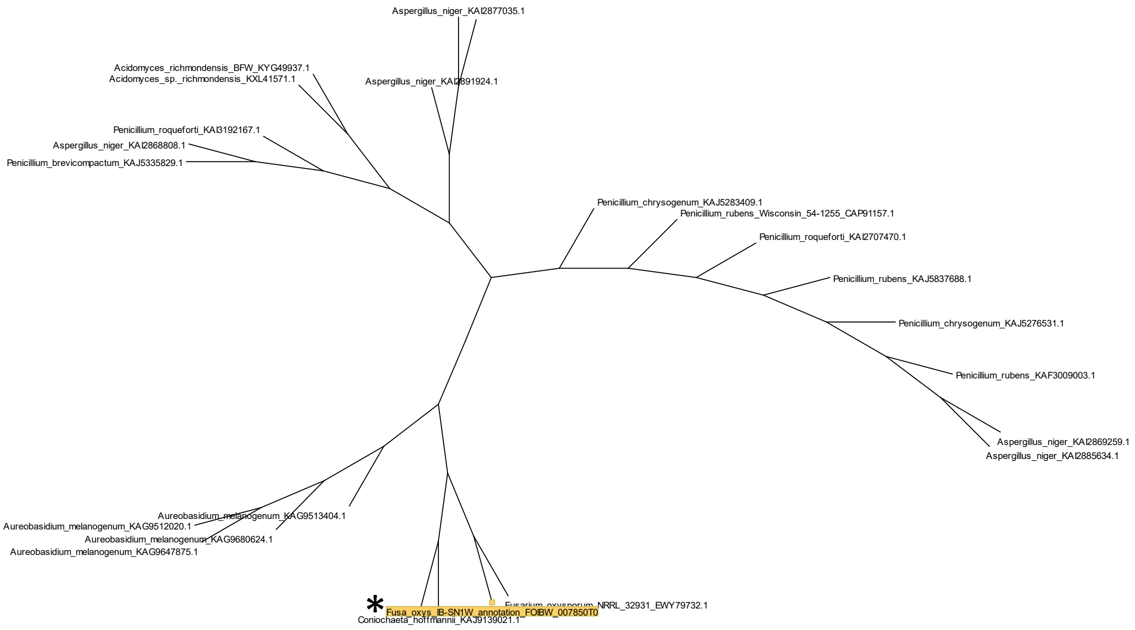

Supplementary Figure 5. Phylogenetic tree of ceruloplasmin homologs. Highlighted in yellow are peptides from IB-SN1W. Asterisks denote its localization in the accessory chromosome

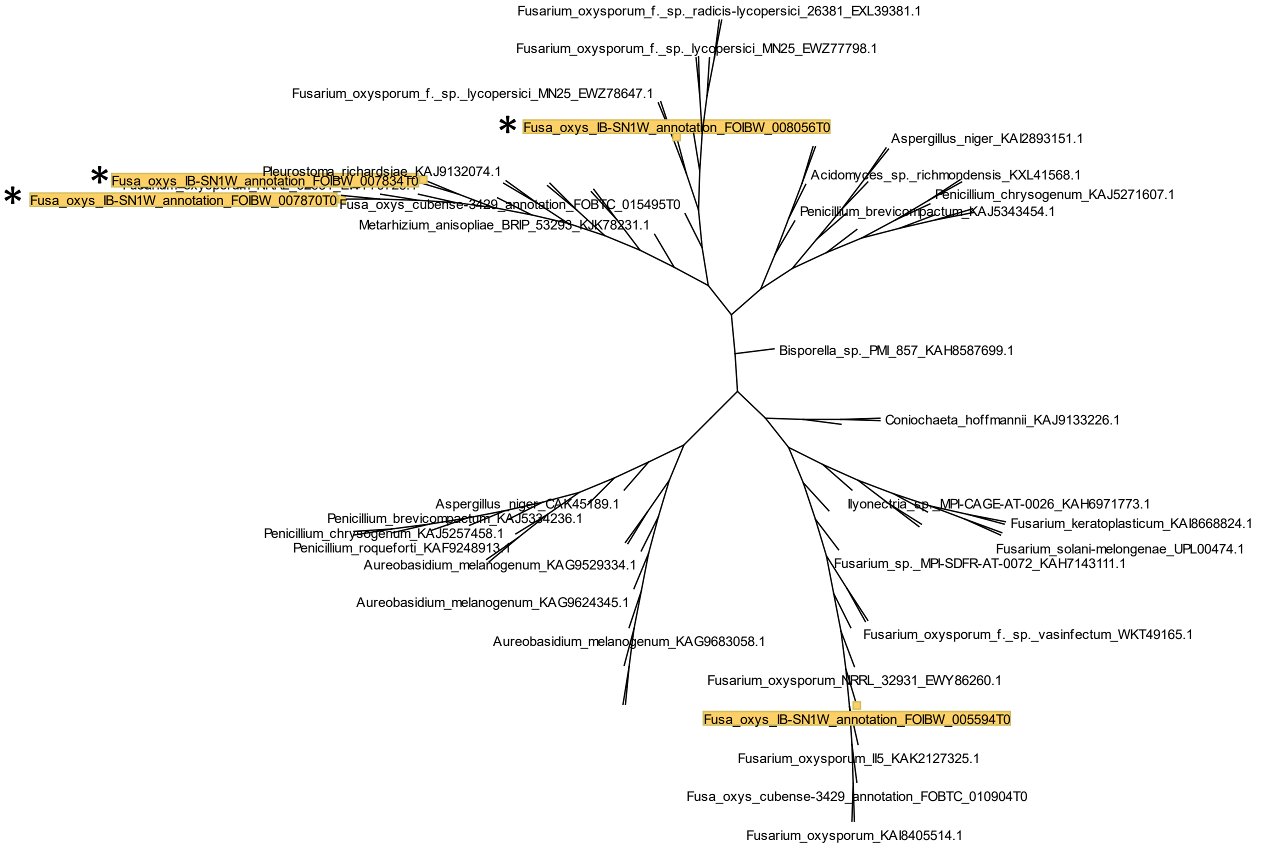

Supplementary Figure 6. Phylogenetic tree of ATX1 homologs. Highlighted in yellow are peptides from IB-SN1W. Asterisks denote its localization in the accessory chromosome

Supplementary Table 1. Accessions used to look for homologs of copper related genes using for orthofinder

**Organism Scientific Name**	**Strain**	**Assembly Accession**	**Gene Count**
Acidomyces richmondensis BFW	BFW	GCA_001592465.1	11177
Acidomyces sp. 'richmondensis'		GCA_001572075.1	10338
Aspergillus niger		GCA_000002855.2	14532
Aspergillus niger	CBS 630.78	GCA_023134295.1	11360
Aspergillus niger	CBS 118.52	GCA_023134325.1	11540
Aspergillus niger	CBS 131.52	GCA_023134385.1	11468
Aureobasidium melanogenum	EXF-10372	GCA_019924015.1	10428
Aureobasidium melanogenum	EXF-9937	GCA_019924185.1	23004
Aureobasidium melanogenum	EXF-9911	GCA_019924195.1	20285
Aureobasidium melanogenum CBS 110374	CBS 110374	GCA_000721775.1	10584
Bisporella sp. PMI 857	PMI 857	GCA_021307315.1	18044
Coniochaeta hoffmannii	EXF-13287	GCA_030052785.1	10382
Coniochaeta ligniaria NRRL 30616	NRRL 30616	GCA_001879275.1	13920
Fusarium decemcellulare	Babe19	GCA_027627305.1	17201
Fusarium keratoplasticum	Fu6.1	GCA_025433545.1	14935
Fusarium keratoplasticum	LHS11.1	GCA_025433555.1	15051
Fusarium oxysporum	Fo5176	GCA_025331925.1	21683
Fusarium oxysporum f. sp. cepae	FoC Fus2	GCA_003615085.1	18852
Fusarium oxysporum f. sp. conglutinans	Fo5176	GCA_014154955.1	17912
Fusarium oxysporum f. sp. lycopersici MN25	MN25	GCA_000259975.2	18297
Fusarium oxysporum f. sp. radicis-cucumerinum	Forc016	GCA_001702695.2	16795
Fusarium oxysporum f. sp. radicis-lycopersici 26381	26381	GCA_000260155.3	18610
Fusarium oxysporum f. sp. vasinfectum	ME23	GCA_030719095.1	16610
Fusarium oxysporum II5	II5	GCA_031834405.1	16335
Fusarium oxysporum NRRL 32931	NRRL 32931	GCA_000271745.2	17642
Fusarium solani-melongenae	CRI 24-3	GCA_023101225.1	15320
Fusarium sp. LHS14.1	LHS14.1	GCA_025433615.1	15935
Fusarium sp. MPI-SDFR-AT-0072	MPI-SDFR-AT-0072	GCA_020744335.1	16026
Ilyonectria sp. MPI-CAGE-AT-0026	MPI-CAGE-AT-0026	GCA_020744155.1	21075
Metarhizium anisopliae BRIP 53293	BRIP 53293	GCA_000426965.1	11415
Penicillium brevicompactum	IBT 35665	GCA_028827555.1	12421
Penicillium chrysogenum	IBT 19737	GCA_028826945.1	12548
Penicillium chrysogenum	IBT 3361	GCA_028827015.1	12254
Penicillium chrysogenum	IBT 35668	GCA_028827035.1	11981
Penicillium roqueforti	LCP96 04111	GCA_015533775.1	9762
Penicillium roqueforti	CBS 147354	GCA_023065465.1	9945
Penicillium roqueforti	CBS 147311	GCA_023141205.1	10313
Penicillium rubens	43M1	GCA_011058885.1	11672
Penicillium rubens	IBT 27055	GCA_028828025.1	11636
Penicillium rubens Wisconsin 54-1255	Wisconsin 54-1255	GCA_000226395.1	13933
Pleurostoma richardsiae	EXF-13308	GCA_030052745.1	12540
Purpureocillium lilacinum	PLFJ-1	GCA_001653265.1	11850
Purpureocillium lilacinum	CBS 150709	GCA_033847395.1	14420
